# Regulatory Network and Prognostic Effect Investigation of PIP4K2A in Leukemia and Solid Cancers

**DOI:** 10.3389/fgene.2018.00721

**Published:** 2019-01-15

**Authors:** Shouyue Zhang, Zhaozhi Li, Xinyu Yan, Li Bao, Yun Deng, Feier Zeng, Peiqi Wang, Jianhui Zhu, Dandan Yin, Fei Liao, Xueyan Zhou, Duyu Zhang, Xuyang Xia, Hong Wang, Xue Yang, Wanhua Zhang, Hu Gao, Wei Zhang, Li Yang, Qianqian Hou, Heng Xu, Yan Zhang, Yang Shu, Yuelan Wang

**Affiliations:** ^1^Department of Thoracic Oncology, Cancer Center, State Key Laboratory of Biotherapy, West China Hospital, Sichuan University, Chengdu, China; ^2^Department of Laboratory Medicine, Precision Medicine Center, State Key Laboratory of Biotherapy and Precision Medicine Key Laboratory of Sichuan Province, West China Hospital, Sichuan University and Collaborative Innovation Center, Chengdu, China; ^3^Key Laboratory of Bio-Resources and Eco-Environment, College of Life Sciences, Sichuan University, Chengdu, China; ^4^State Key Laboratory of Oral Diseases, West China Hospital of Stomatology, Sichuan University, Chengdu, China; ^5^Integrated Biomedical Sciences Program, University of Tennessee Health Science Center, Memphis, TN, United States; ^6^Department of Hematology and Hematology Research Laboratory, Sichuan University, Chengdu, China; ^7^Department of Emergency, West China Second University Hospital, Sichuan University, Chengdu; ^8^Department of Clinical Pharmacology, Hunan Key Laboratory of Pharmacogenetics, Xiangya Hospital, Central South University, Changsha, China

**Keywords:** acute lymphoblastic leukemia, cancer, TCGA, TFDP1, PIP4K2A

## Abstract

Germline variants of *PIP4K2A* impact susceptibility of acute lymphoblastic leukemia (ALL) through inducing its overexpression. Although limited reports suggested the oncogenic role of *PIP4K2A* in cancers, regulatory network and prognostic effect of this gene remains poorly understood in tumorigenesis and leukemogenesis. In this study, we conducted genome-wide gene expression association analyses in pediatric B-ALL cohorts to discover expression associated genes and pathways, which is followed by the bioinformatics analyses to investigate the prognostic role of *PIP4K2A* and its related genes in multiple cancer types. 214 candidates were identified to be significantly associated with *PIP4K2A* expression in ALL patients, with known cancer-related genes rankings the top (e.g., *RAC2*, *RBL2*, and *TFDP1*). These candidates do not only tend to be clustered in the same types of leukemia, but can also separate the patients into novel molecular subtypes. *PIP4K2A* is noticed to be frequently overexpressed in multiple other types of leukemia and solid cancers from cancer cohorts including TCGA, and associated with its candidates in subtype-specific and cancer-specific manners. Interestingly, the association status varied in tumors compared to their matched normal tissues. Moreover, *PIP4K2A* and its related candidates exhibit stage-independent prognostic effects in multiple cancers, mostly with its lower expression significantly associated with longer overall survival (*p* < 0.05). Our findings reveal the transcriptional regulatory network of *PIP4K2A* in leukemia, and suggest its potentially important role on molecular subtypes of multiple cancers and subsequent treatment outcomes.

## Introduction

Acute lymphoblastic leukemia (ALL) is the most common pediatric malignancy (Greaves, 2006; Pui and Evans, 2006), induced through multifactorial mechanisms involving both environmental and genetic factors (Buffler et al., 2005). A series of genome-wide association studies (GWASs) have identified several common risk loci for genetic predisposition basis of ALL in several genes (e.g., *ARID5B, IKZF1, CEBPE, CDKN2A, PIP4K2A-BMI1*, and *GATA3*) (Papaemmanuil et al., 2009; Trevino et al., 2009; Sherborne et al., 2010; Migliorini et al., 2013; Perez-Andreu et al., 2013; Xu et al., 2013; Perez-Andreu et al., 2015; Xu et al., 2015). As more and more causal variants are considered to impact on gene expression rather than inducing amino acid changes, analyses for expression quantitative trait loci (eQTL) in ALL have also been conducted. For instance, *PIP4K2A* expression is strongly impacted by the genotypes of its adjacent single nucleotide polymorphisms (SNPs), which have been validated in several independent patient cohorts for ALL susceptibility (Walsh et al., 2013; Liao et al., 2016), suggesting *PIP4K2A* SNPs may play role on leukemogenesis through inducing its overexpression. Meanwhile, current evidence has also shown a ethnicity-specific and subtype-specific correlation between *PIP4K2A* SNPs and ALL susceptibility (Migliorini et al., 2013; Walsh et al., 2013; Xu et al., 2013), which is similar as *ARID5B* SNPs (Xu et al., 2012, 2013). However, how *PIP4K2A* contributes to leukemogenesis of ALL and its subtypes remains to be poorly understood.

*PIP4K2A* encodes a major component of phosphatidylinositol 5-phosphate 4-kinase type-II (Rameh et al., 1997). As actively expressed in peripheral blood cells (Divecha et al., 1995), *PIP4K2A* may facilitate the conversion of phosphorylation of phosphatidylinositol 5-phosphate (PtdIns5P) to generate various phosphoinositide species, which regulate crucial cellular functions including proliferation, survival, glucose uptake and migration (Fiume et al., 2015). A few reports have demonstrated that *PIP4K2A* may play a critical role on development and cellular activities maintaining. For instance, *PIP4K2A* expression can influence cell response to oxidative stress by regulating the level of PtdIns5P (Wilcox and Hinchliffe, 2008; Jones et al., 2013). Depletion of *PIP4K2A* is associated with decreased mTOR signaling, and induces developmental defects in Drosophila and zebrafish (Elouarrat et al., 2013; Gupta et al., 2013). Besides, PIP4K2A is considered to control neuronal KCNQ channels, disorders of which are implicated in schizophrenia (Fedorenko et al., 2008, 2009), and may also involve in insulin signaling by regulating PI3/AKT pathway. Knocking out of *PIP4K2B*, another component of phosphatidylinositol 5-phosphate 4-kinase type-II, can increase insulin sensitivity and reduce adiposity (Carricaburu et al., 2003; Lamia et al., 2004). However, the role of *PIP4K2A* on tumorigenesis (including leukemogenesis) has been mentioned in limited reports. Recently, a cell-line based experiment indicates that knockdown of *PIP4K2A* in acute myeloid leukemia (AML) results in accumulation of the cyclin-dependent kinase inhibitors (i.e., CDKN1A and CDKN1B), G1 cell cycle arrest and apoptosis (Jude et al., 2015). Nevertheless, *PIP4K2A* knockdown in normal hematopoietic stem cells and progenitor cells doesn’t impair cell growth. Since knockdown of *PIP4K2A* only exerts inhibitory effects on tumor cells, this selective effect implies that *PIP4K2A* might be a candidate target for cancer treatment. In fact, *PIP4K2A* is noted to be up-regulated in breast tumor tissue and high expression of *PIP4K2A* is correlated with loss of p53. Knockdown of both *PIP4K2A* and *PIP4K2B* in a p53-deficient breast cell line dramatically abrogates cell proliferation (Emerling et al., 2013). This could be explained by impaired glucose metabolism and consequently increased reactive oxygen species (ROS), which leads to senescence. Also, *PIP4K2A* is considered to impact on sensitivity of some cancer drugs (e.g., paclitaxel), thus may be associated with overall survival of the treated cancer patients (Niu et al., 2012).

Single nucleotide polymorphisms around *PIP4K2A* are associated with ALL susceptibility through impacting expression level of *PIP4K2A*, and accumulating evidence indicates the potential role of *PIP4K2A* on cancer cells, however, it is poorly understood that how *PIP4K2A* impact on tumorigenesis (including leukemogenesis). In this study, we conducted array based genome-wide gene expression association analyses to investigate the potential regulatory network of *PIP4K2A* in leukemia, which is proved to be efficient in our previous work (Hou et al., 2017). Besides, we will also systematically examine the *PIP4K2A* status in multiple cancers and evaluate its prognostic effect.

## Results

### Screening of PIP4K2A-Related Genes in B-ALL and Their Association Status in B-ALL Subtypes

As the expression of *PIP4K2A* was considered to impact on leukemogenesis, we thus downloaded array-based expression data of eight independent pediatric B-ALL patient cohorts from public resource (Supplementary Table S1), and evaluated the expression correlation of *PIP4K2A* with the rest of other genes using a linear regression model. A series of filter criteria (e.g., *p-*value cutoff, *r*^2^, and correlation direction) were applied to determine more reliable *PIP4K2A*-related candidates for regulation network construction (Supplementary Figure S1). Altogether, 234 probes in 214 genes were significantly correlated to *PIP4K2A* expression with *p* ≤ 0.05 in all patient cohorts in the same direction (Supplementary Figure S1 and Supplementary Table S2). Through a stricter filter (i.e., *p* ≤ 2 × 10^-6^ and association *r*^2^ ≥ 0.1 in at least five cohorts), 23 genes were eventually identified as the strongest candidates (Supplementary Table S3).

**FIGURE 1:**
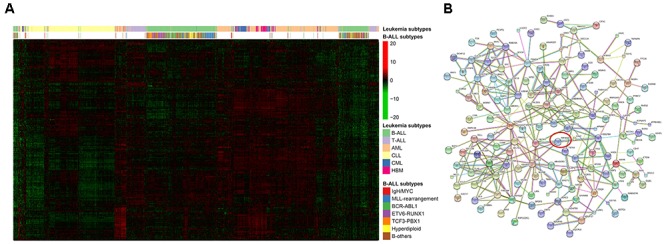
Regulatory statement of *PIP4K2A* and its related candidates in leukemia. **(A)** Expression clustering of PIP4K2A-related genes, with leukemia types and B-ALL subtypes were labeled. **(B)** Protein–protein interaction network of *PIP4K2A*-related genes. Line thickness indicates the strength of data support, and nodes disconnected with the main network were hidden.

Since *PIP4K2A* is a major component phosphate kinase, we sought to reveal its regulatory network by focusing on 10 transcription factors we screened out (Table 1). For instance, *RBL2* is a member of retinoblastoma-susceptibility (RB) anti-oncogene family, which regulates cell cycle through inhibiting E2F-mediated transcription. Dysfunction of this gene is commonly involved in evolution of ALL (Ahuja et al., 1991; Stock et al., 2000; Schmitz et al., 2005), thus indicating a potentially significant role of *RBL2* on B-ALL development. Pathway analysis was further conducted using DAVID Functional Annotation Tools, and three annotation clusters of gene sets exhibited significance with at least one term having Bonferroni *p*-value < 0.05 (Supplementary Table S4), including leukocyte migration pathway. Additionally, we illustrated the interactions between candidates by constructing a protein–protein interaction network based on STRING (Figure 1B), and identified multiple *PIP4K2A* related proteins, including the top gene of *RAC2*, which is highly expressed in immune organs. Since *RAC2* belongs to the Rho small GTPase subfamily, which can regulate cell movement, proliferation, and survival, involvement of *PIP4K2A* in leukemogenesis may be partially explained by their interactions. In this part, we firstly built the regulatory network of *PIP4K2A* in B-ALL.

**Table 1:** Candidates of *PIP4K2A* related DNA-dependent transcriptional regulatory genes in different dataset of B-ALL.

Probe ID		202098_s_at	202449_s_at	204529_s_at	205038_at	209107_x_at	209930_s_at	210249_s_at	212330_at	212331_at	216241_s_at	43544_at
**Gene**		***PRMT2***	***RXRA***	***TOX***	***IKZF1***	***NCOA1***	***NFE2***	***NCOA1***	***TFDP1***	***RBL2***	***TCEA1***	***MED16***
GSE7440 (*n* = 99)	*p*-Value	7.49 × 10^-10^	7.10 × 10^-13^	8.58 × 10^-7^	4.89 × 10^-5^	1.69 × 10^-10^	2.64 × 10^-9^	2.93 × 10^-13^	2.94 × 10^-12^	8.99 × 10^-10^	8.56 × 10^-8^	3.64 × 10^-11^
	Coefficient	0.64	0.61	0.33	0.38	0.84	0.38	0.92	0.51	0.58	0.65	0.74
	se	0.09	0.07	0.06	0.09	0.12	0.06	0.11	0.06	0.09	0.11	0.10
	Adjusted *r*^2^	0.32	0.41	0.21	0.15	0.34	0.30	0.42	0.39	0.32	0.25	0.36
GSE10255 (*n* = 161)	*p*-Value	0.01	0.01	7.56 × 10^-4^	0.03	4.10 × 10^-3^	4.80 × 10^-15^	1.84 × 10^-3^	1.35 × 10^-3^	4.50 × 10^-3^	3.65 × 10^-3^	9.86 × 10^-9^
	Coefficient	0.40	0.20	0.16	0.26	0.57	0.26	0.51	0.15	0.37	0.49	0.72
	se	0.15	0.08	0.05	0.12	0.20	0.03	0.16	0.05	0.13	0.17	0.12
	Adjusted *r*^2^	0.04	0.03	0.06	0.02	0.04	0.32	0.05	0.06	0.04	0.05	0.18
GSE10792 (*n* = 160)	*p*-Value	5.74 × 10^-7^	0.01	0.02	0.03	0.02	1.67 × 10^-3^	0.04	8.94 × 10^-7^	6.12 × 10^-4^	2.96 × 10^-3^	5.79 × 10^-4^
	Coefficient	0.53	0.22	0.16	0.33	0.48	0.24	0.44	0.29	0.64	0.84	0.51
	se	0.10	0.08	0.07	0.14	0.21	0.07	0.21	0.06	0.18	0.28	0.14
	Adjusted *r*^2^	0.26	0.07	0.05	0.05	0.05	0.11	0.04	0.26	0.13	0.10	0.13
GSE11877 (*n* = 207)	*p*-Value	4.93 × 10^-9^	1.65 × 10^-3^	2.19 × 10^-3^	1.03 × 10^-6^	1.02 × 10^-4^	1.60 × 10^-5^	1.21 × 10^-4^	2.24 × 10^-3^	8.65 × 10^-13^	1.96 × 10^-4^	2.71 × 10^-7^
	Coefficient	0.39	0.17	0.17	0.33	0.49	0.15	0.46	0.20	0.59	0.43	0.34
	se	0.06	0.05	0.05	0.07	0.12	0.03	0.12	0.06	0.08	0.11	0.06
	Adjusted *r*^2^	0.15	0.04	0.04	0.11	0.07	0.08	0.07	0.04	0.22	0.06	0.12
GSE13351 (*n* = 107)	*p*-Value	4.53 × 10^-9^	3.81 × 10^-4^	3.94 × 10^-3^	1.02 × 10^-3^	1.80 × 10^-5^	1.89 × 10^-6^	3.54 × 10^-5^	1.72 × 10^-3^	3.46 × 10^-12^	4.43 × 10^-5^	9.53 × 10^-6^
	Coefficient	0.58	0.24	0.19	0.31	0.91	0.28	0.82	0.20	0.67	0.58	0.45
	se	0.09	0.07	0.07	0.09	0.20	0.06	0.19	0.06	0.08	0.14	0.10
	Adjusted *r*^2^	0.31	0.12	0.08	0.10	0.18	0.22	0.16	0.09	0.41	0.16	0.19
GSE13425 (*n* = 190)	*p*-Value	9.14 × 10^-5^	1.01 × 10^-6^	5.30 × 10^-3^	1.18 × 10^-3^	2.07 × 10^-4^	2.99 × 10^-3^	3.79 × 10^-3^	5.78 × 10^-3^	1.32 × 10^-6^	3.31 × 10^-4^	1.47 × 10^-3^
	Coefficient	0.36	0.39	0.25	0.31	0.66	0.18	0.52	0.20	0.54	0.54	0.33
	se	0.09	0.08	0.09	0.09	0.17	0.06	0.18	0.07	0.11	0.15	0.10
	Adjusted *r*^2^	0.09	0.14	0.04	0.06	0.08	0.05	0.05	0.04	0.14	0.08	0.06
GSE33315 (*n* = 575)	*p*-Value	4.32 × 10^-17^	9.08 × 10^-5^	3.21 × 10^-3^	1.88 × 10^-9^	1.38 × 10^-12^	1.32 × 10^-26^	8.79 × 10^-14^	3.64 × 10^-16^	1.81 × 10^-23^	8.81 × 10^-11^	5.83 × 10^-21^
	Coefficient	0.56	0.16	0.11	0.34	0.75	0.22	0.67	0.24	0.68	0.57	0.60
	se	0.06	0.04	0.04	0.05	0.10	0.02	0.09	0.03	0.06	0.09	0.06
	Adjusted *r*^2^	0.13	0.03	0.02	0.07	0.10	0.21	0.11	0.13	0.19	0.08	0.17
GSE635 (*n* = 173)	*p*-Value	2.01 × 10^-6^	1.38 × 10^-4^	5.41 × 10^-7^	0.02	3.65 × 10^-3^	1.23 × 10^-3^	2.97 × 10^-3^	2.86 × 10^-3^	3.44 × 10^-5^	6.07 × 10^-5^	1.01 × 10^-3^
	Coefficient	0.41	0.28	0.29	0.20	0.51	0.14	0.49	0.18	0.44	0.64	0.30
	se	0.08	0.07	0.05	0.09	0.17	0.04	0.16	0.06	0.10	0.16	0.09
	Adjusted *r*^2^	0.12	0.08	0.13	0.02	0.04	0.05	0.04	0.05	0.09	0.08	0.06


**FIGURE 2:**
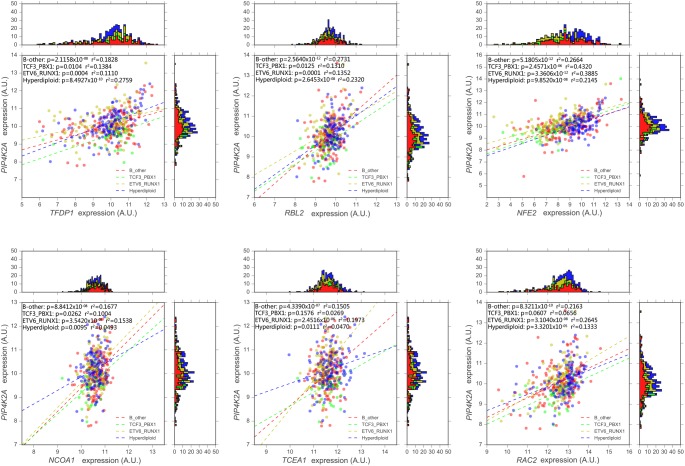
Expression association status of *PIP4K2A* with some of important candidates in different subtypes of B-ALL (i.e., B-others, ETV6-RUNX1, hyperdiploid, and TCF3-PBX1) in the largest available pediatric B-ALL cohort (i.e., GSE33315).

Next, the association between *PIP4K2A* and its candidates were investigated in different molecular subtypes of the largest pediatric B-ALL cohort with available subtype information (GSE33315) (Supplementary Table S5). To exclude the impact of small sample size of some subtypes, such as BCR-ABL and MLL rearrangement, we mainly focused on four large patient groups, including hyperdiploid, ETV6-RUNX1, TCF3-PBX1 molecular subtypes as well as B-others. Totally 85 out of 214 genes are significantly associated with *PIP4K2A* expression in all four patient groups with the same direction, including seven transcription factors (Table 1 and Figure 2). Although similar association status was observed in different subtypes in terms of *r*^2^ and coefficients of these transcription factors (e.g., *TFDP1*, *RBL2*, and *NFE2*), subtype-specificity was also noticed. For instance, *NCOA1* is significantly associated with *PIP4K2A* in all four patient groups with varied coefficients in different subtypes, while *TCEA1* is even unrelated to *PIP4K2A* in TCF3-RUNX1 subtypes, indicating regulation network of *PIP4K2A* may vary in subtype-specific manner.

### Varied Association Status of PIP4K2A With Candidate Genes in Different Leukemia Types

**FIGURE 3:**
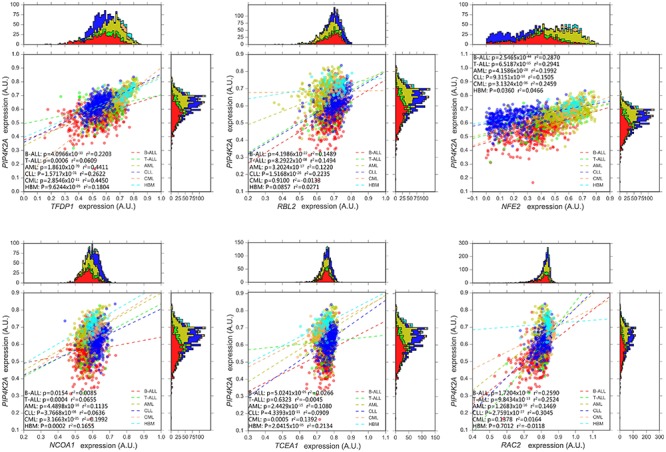
Expression association status of *PIP4K2A* with some of important candidates in different types of leukemia, including B-ALL (*N* = 576), T-ALL (*N* = 174), AML (*N* = 542), CLL (*N* = 448), CML (*N* = 76), and healthy bone marrow (HBM, *N* = 74) based on GSE13204.

Next, dataset GSE13204 (3248), which contains a large number of patients with B-ALL, T-ALL, AML, chronic lymphoblastic leukemia (CLL), chronic myeloid leukemia (CML) as well as healthy bone marrow (HBM), was selected for validation and further analyses. Expression of all *PIP4K2A*-related genes in B-ALL tends to be clustered in terms of leukemia types, illustrating distinct expression patterns of these candidates in different leukemia types and their different roles on leukemogenesis (Figure 1A). Not surprisingly, 99.07% (212/214) of the candidates were significantly associated with *PIP4K2A* expression in B-ALL of this cohort with the same direction, proving the reliability of their correlations. Importantly, the consistent rate for significant association between *PIP4K2A* and its candidates declined to 96.26% (206/214), 91.1% (195/214), 87.85% (188/214), 66.36% (142/214) and 49.53% (106/214) in CLL, AML, T-ALL, CML and HBM, respectively, even with opposite association directions to that in B-ALL for some genes, which account for 0.97% (2/206), 3.08% (6/195), 0% (0/188), 2.11% (3/142) and 4.72% (5/106) of in CLL, AML, T-ALL, CML and HBM, respectively (Supplementary Table S6). While the transcription factors are more stable, with higher consistent rates of significant association and no opposite direction (Figure 3). Above results implies that the association of PIP4K2A with its candidates discovered in B-ALL may also exists in other leukemia types at different levels. Notably, association status of *PIP4K2A* and its candidates exhibit the lowest consistent rate in health bone marrow in terms of *p*-value as well as *r*^2^ (Supplementary Table S6), indicating the dysfunction of PIP4K2A regulation network in leukemia compared to normal tissues.

### PIP4K2A Expression Profile and Potential Genes Associated With PIP4K2A Expression in Multiple Cancer Types

Although higher *PIP4K2A* expression may contribute to higher risk of leukemogenesis, normal B cells are unavailable as matched control for further analyses. Therefore, we further investigated the expression profile of *PIP4K2A* and its associated genes in other cancer types with paired tumor/normal tissues from The Cancer Genome Atlas (TCGA) (Supplementary Figure S6). With implicated expression information in multiple types of cancers, up to 8% of patients exhibited up-regulated *PIP4K2A* in tumors compared with their matched normal tissues in all available cancer types except a few down-regulations (e.g., KIRC) (Supplementary Figure S2), suggesting the potential widespread oncogenic role of *PIP4K2A* in tumorigenesis apart from leukemia.

**FIGURE 4:**
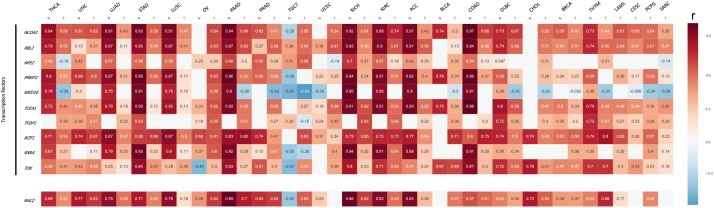
Expression association status of *PIP4K2A* with 10 transcriptional regulators in different cancer types of TCGA. Heatmap of *p*-value and *r*^2^ of correlation of *PIP4K2A*-related transcriptional regulators with normal and tumor tissues of 23 cancer types. White box indicates non-significant (*p-*value > 0.05).

Next, we downloaded expression data from TCGA to investigate the association of *PIP4K2A* with its associated transcription factors in tumors and normal separately in each cancer type (Supplementary Tables S7, S8), Among the 23 types of cancers each with at least 100 patients having available follow-up information, nearly 70% of these transcription factors exhibit consistent association in both tumor and normal tissues, yet with varied coefficients and *r*^2^, even opposite directions (Figure 4). Interestingly, tumor-specific or control-specific associations were noticed. The candidates exhibited stronger association in normal tissues (e.g., normal tissues of KIRP, KICH, KIRC, ACC, and COAD) than tumors, whereas at least half of genes lost their correlation, or even turn to be negatively associated with *PIP4K2A* preferentially in normal tissues of OV, TGCT, UCEC, BLCA, and CHOL. For instance, *NCOA1* is more correlated to *PIP4K2A* in normal tissues than that in tumors in terms of *r*^2^. The correlations of *PIP4K2A* with *TFDP1* tend to exhibit tumor-specific (i.e., significant in 21/23 tumors but 9/20 normal tissues) (Figure 4 and Supplementary Figure S3), while that with *MED16* is tend to be in control-specific manner (i.e., significant in 14/23 tumors but 15/20 normal tissues) (Figure 4). Additionally, opposite association directions of tumor vs. normal were also observed, such as *MED16* in THCA (Figure 4), indicating the varied regulatory status and role of *PIP4K2A* on tumor vs. normal tissues in different types of cancers.

### Impact of PIP4K2A and Its Correlated Gene on Overall Survival Rate

**FIGURE 5:**
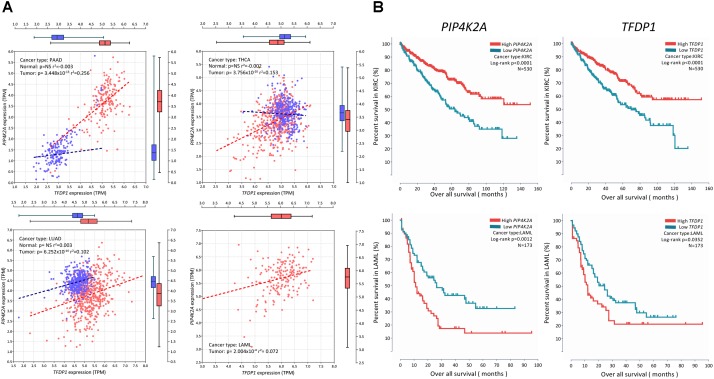
Correlation of *PIP4K2A* and its related candidates with survival status in different cancer types. **(A)** Expression association status of *TFDP1* with *PIP4K2A* in LAML and KIRC; **(B)** association status of *TFDP1* and *PIP4K2A* with overall survival in LAML and KIRC.

Due to the potentially important role of *PIP4K2A* in tumors, we further explored the possible role of *PIP4K2A* expression on survival rate in different cancer types. We focused on the transcription factors because of their important roles on *PIP4K2A* network described above. Totally, significant correlations were observed in 6 out of 23 cancer types (i.e., BLCA, KIRC, LAML, LIHC, SARC, and STAD) (Supplementary Figure S4). In particular, lower expression of *PIP4K2A* in tumors is associated with longer overall survival in all these cancer types except KIRC (Figure 5), suggesting the potential prognostic role of *PIP4K2A* overexpression in multiple cancer types, which is frequently observed as described above. Moreover, we also evaluated the correlation of survival status in these six cancer types with the expression levels of *PIP4K2A* related transcription factors (Supplementary Table S9). Strikingly, *TFDP1* exhibits the strongest consistency with *PIP4K2A* in the same direction in five out of six cancer types (i.e., KIRC, LAML, SARC, BLCA, and LIHC) but not STAD (Supplementary Figure S4 and Supplementary Table S9). On the contrary, such consistency is lower for other transcription factors. For non-transcription factors, an opposite direction was even identified, such as *RAC2* (Supplementary Figure S4 and Supplementary Table S9). Hence, *TFDP1* may play an important role on the transcriptional regulatory network of *PIP4K2A* to impact on the survival rate in multiple cancer types.

## Discussion

*PIP4K2A* is largely implicated in basal cellular activities such as proliferation, survival, glucose uptake and migration, and plays an essential role for tumor growth. Due to extensive expression and pleiotropic effect of *PIP4K2A* in multiple tissues, it is important to unravel the transcriptional regulatory network of *PIP4K2A* in different cancer types, which is yet time/effect-consuming. Based on the assumption that genes participating in the same regulatory network are associated in terms of expression level, and closer transcriptional factors exhibit more significant correlation, it will be much easier to screen potential *PIP4K2A*-related genes by genome-wide gene expression association analyses by using available public microarray datasets. A series of GWASs and subsequent validations have revealed that SNPs in *PIP4K2A* locus are significantly associated with ALL susceptibility, possibly through impact on *PIP4K2A* expression, indicating the potential role of *PIP4K2A* over-expression in leukemogenesis. Therefore, 214 genes were identified to be associated with *PIP4K2A* expression with the similar pipeline we used previously (Hou et al., 2017). Although association strength varied in different types of leukemia, or even subtypes of B-ALLs, almost all of these candidates can be validated in B-ALL of another big patient cohort, while a large proportion of them can also be validated in other types of leukemia in terms of expression association. These findings strongly indicate the consistency and stability of *PIP4K2A* network. Since *PIP4K2A* encodes a kinase, it is desirable to focus on characteristic regulatory network of *PIP4K2A* in different B-ALL subtypes, respectively, by analyzing larger samples. Additionally, the over-expression of *PIP4K2A* could be influenced by the chemotherapeutic treatment applied, so we have searched all the datasets to see if the influence of chemotherapeutic treatment existed in these cohorts. Among eight patient cohorts analyzed in Table 1, the patients from GSE7440 and GSE11877 received chemotherapy, while the patients from GSE10255 and GSE13425 were newly diagnosed with ALL without any initial therapy. Other cohorts didn’t tell what treatment the patients received. Therefore, we compared the association status of transcription factors with *PIP4K2A* in abovementioned four cohorts. It turns out that no significant difference in correlation exists between the cohorts who received chemotherapy and those who did not, indicating that the chemotherapeutic treatment does not significantly influence the association between *PIP4K2A* and candidates, at least in the analyzed cohorts.

Noteworthily, our findings identified some genes that have been reported to be related to leukemogenesis or ALL development. As one of the strongest *PIP4K2A*-related candidates, the transcription factor *RBL2* belongs to retinoblastoma-susceptibility (RB) anti-oncogene family. The RB gene is an anti-oncogene regulating cell cycle through inhibiting E2F-mediated transcription, which is frequently altered and thus quite commonly involved in evolution of ALL (Ahuja et al., 1991; Stock et al., 2000; Schmitz et al., 2005). *RBL2* demonstrates statistically significant and positive correlation with *PIP4K2A* expression in four relatively larger samples of B-ALL subtypes (i.e., hyperdiploid, ETV6-RUNX1, B-other and TCF3-PBX1 subtype). *RBL2* also exhibits significant correlation exclusively in tumor tissues of OV, BLCA, and CHOL, indicating a cancer type-specific correlation between *RBL2* and *PIP4K2A*. Another example is *TFDP1*, this gene exhibits extraordinarily strong association with *PIP4K2A*. Same as *RBL2*, *TFDP1* also shows evident association with *PIP4K2A* in four large samples of B-ALL subtypes as well as all types of leukemia. And in 23 other cancer types, *TFDP1* remains the significance in 21 types of cancer. Specifically, in KIRC and PAAD, *PIP4K2A* expression is prominently affected by *TFDP1*. Besides, the impact of *PIP4K2A* and *TFDP1* expression on survival status in various cancers demonstrates great consistency, and these two genes are also significantly correlated in these cancers. Above findings reveal quite close interactions between *PIP4K2A* and *TFDP1* as well as the important role of *TFDP1* in regulatory network of *PIP4K2A*. However, no clear binding motif information is available due to the limited studies on *TFDP1*. Some other candidates are linked to *PIP4K2A* according to PPI prediction, and we considered it is appropriate to start with these leukemia-related or cancer-related genes in order to reveal the transcriptional regulatory network of *PIP4K2A* in B-ALL. With the limited database, some of the candidate transcription factors have the predicted binding sites in *PIP4K2A* promoter or enhancer region, including *RXRA* (Supplementary Figure S5 and Supplementary Table S10). However, further experimental analyses are largely needed to test whether *PIP4K2A* is regulated by these transcription factors through direct binding. Unfortunately, we didn’t screen out the well-known genes in mTOR pathway or KCNQ channel, probably because *PIP4K2A* may be involved in these pathways through post-transcriptional regulation or protein interactions, which can’t be detected through expression association approaches.

Importantly, the candidate transcription factors are also significant associated with *PIP4K2A* expression in multiple cancer types apart from leukemia. Generally, candidate genes demonstrate much stronger association in normal tissues than tumors. Among 10 transcription factors, some genes exhibit significant associations in all 23 cancer types such as *RBL2* and *IKZF1*, indicating their important role on *PIP4K2A* and ubiquitous interactions with *PIP4K2A* in the regulatory network among different cancers, as well as the reliability of our screening process. Interestingly, *IKZF1* is one of most important transcription factors involved in hematopoietic development, mutations and deletions of this gene are frequently observed in leukemia cells of B-ALL patients. Meanwhile, SNPs in *IKZF1* are also significantly associated with ALL susceptibility across multiple ethnicities, probably through impacting on the expression level of *IKZF1*. Therefore, the expression correlation of *IKZF1* with *PIP4K2A* indicates that these two genes may interact with each other to facilitate leukemogenesis as well as tumorigenesis. Although insignificant in some cancer types, more than 70% of associations between *PIP4K2A* and the rest of the transcription factors exhibit statically significant, including cancer-specific or normal-specific manner. Moreover, loss or even opposite direction of correlations were observed in quite a few genes between tumor and normal tissues, suggesting tumor-specific dysfunction of *PIP4K2A* regulatory network may contribute to tumorigenesis.

Due to the lack of survival information of the ALL patients, we can’t estimate the impact of SNP genotypes and subsequent SNP-induced expression changes on survival rate. However, we noticed that *PIP4K2A* overexpression is related to worse treatment outcomes of multiple cancer types of TCGA, except higher *PIP4K2A* expression related to better survival status in KIRC. Consistently, down-regulation of *PIP4K2A* in tumors is much more frequently observed in KIRC than any other types of cancer in TCGA (Supplementary Figure S2). Not surprisingly, some of the *PIP4K2A*-related genes are also related to survival rate in the same direction, among which *TFDP1* exhibits the highest consistency, suggesting that TFDP1 may interact with *PIP4K2A* to impact on both tumorigenesis as well as treatment outcomes. However, the prognostic value of *PIP4K2A* and its regulatory partners should be evaluated in independent cancer cohorts.

To sum up, we have utilized available public microarray datasets and developed a systematic screening to find expression-based regulatory network of *PIP4K2A*. Because very limited studies have reported to explore the functional role of *PIP4K2A* on leukemogenesis despite of its significant association with ALL susceptibility as well as overexpression in multiple types of cancers, our findings may shed a new light on epigenetic regulation of *PIP4K2A*, and provide a clue for the further functional studies on this gene in cancer.

## Materials and Methods

### Construction of Regulatory Network of PIP4K2A

Expression level of all genes in B-ALL patients were downloaded from Gene Expression Omnibus [GSE7440 (Bhojwani et al., 2008), GSE11877 (Kang et al., 2010), GSE10792 (Bungaro et al., 2009), GSE13351 (Den Boer et al., 2009), GSE13425 (Holleman et al., 2004), GSE635 (Sorich et al., 2008), GSE10255 (Kirschner-Schwabe et al., 2006), GSE4698 (Zhang et al., 2012), GSE33315 (Haferlach et al., 2010), and GSE13204 (Curtis et al., 2012)]. Associations of PIP4K2A expression with all the rest genes were calculated using linear regression model. Afterward, we used multiple steps to conduct candidate screening and determine the strong candidates (Supplementary Figure S1).

All the PIP4K2A-related candidates were imported into the STRING^1^ to show the known protein–protein interaction network (Szklarczyk et al., 2015). We used line thickness to indicate the strength of data support, and the nodes disconnected with the main network were hidden.

Functional enrichment was determined using the DAVID functional annotation tool^2^ (Huang da et al., 2009a,b). The functional categories used were GO term related to biological process (BP), cellular component (CC), and molecular function (MF)^3^. Multiple criteria were applied for pathway analyses of candidates, including *P*-value, FDR, etc. (Huang da et al., 2009b). Various top-related pathways were selected (i.e., lysosome, porphyrin biosynthesis and adhesion, and diapedesis of granulocytes).

With limited information of transcription factors binding motif, we predicted some candidate transcription factors by importing the specific sequence of PIP4K2A in to JASPAR^4^. Some of these transcription factors got high relative scores and had predicted binding sites in PIP4K2A promoter or enhancer regions.

### Exploration of Differences Between PIP4K2A Regulatory Networks in Different Subtypes

We divided the patients into multiple groups according to the subtypes of B-ALL and the subtypes of leukemia from the large two cohorts, GSE33315 and GSE13204, respectively. To study the difference of *PIP4K2A* regulatory networks in various subtypes, we calculated the association of *PIP4K2A* with all the rest genes by linear regression model in diverse subtypes. The proportions of *PIP4K2A*-related candidates in all significant related genes were computed.

We selected 133 *PIP4K2A*-related probes by various criteria (i.e., *p* ≤ 2 × 10^-6^ and association *r*^2^ ≥ 0.1 in at least five cohorts, and *r*^2^ ≥ 0.3 in at least one cohort). Then, the expression levels of these candidates in GSE13204 were used to generate the heatmap, clustering by the subtypes of leukemia and the subtypes of B-ALL, respectively.

### Discovery of Association of PIP4K2A With Its Related Genes in Multiple Cancers and Corresponding Normal Tissues

We compared the expression level of PIP4K2A in various cancer types in COSMIC dataset^5^, and computed the frequency of high-regulated and low-regulated PIP4K2A samples in different status.

Some functional *PIP4K2A*-related transcription factors were picked out using the Animal TFDB database (42)^6^. Gene expression RNAseq [log_2_(tpm+0.001)] of the candidates in 23 TCGA cancers (THCA, LIHC, LUAD, STAD, LUSC, OV, PAAD, PRAD, TGCT, UCEC, KICH, KIRC, ACC, BLCA, COAD, DLBC, CHOL, BRCA, THYM, LAML, CESC, PCPG, SARC) (Supplementary Table S7) and corresponding normal tissues in both TCGA and GTEX were acquired from UCSC Xena (Vivian et al., 2017)^7^. The association of the expression level of the selected transcription factors with *PIP4K2A* was subsequently calculated using linear regression models as well.

To further detect the association of *PIP4K2A* with these genes, the *r*^2^ of *PIP4K2A* and its related transcription factors as well as *RAC2*, that we calculated by linear model as we described above, were imported to generate the heatmap, in which white indicates non-significance.

### Comparison of PIP4K2A and Its Correlated Gene on Overall Survival Rate

The clinical information for survival analyses was also acquired from UCSC Xena, including patient ID, overall survival information, etc. We divided the expression levels of *PIP4K2A*-related transcription factors into two groups, high-expression level and low-expression level, by the significant low point of their histogram, and used this standard to conduct survival analysis in different cancer types.

## Data Availability Statement

Datasets analyzed for this study are available in GEO repository (http://www.ncbi.nlm.nih.gov/gds): GSE635, GSE10792, GSE13351, GSE13425, GSE10255, GSE4698, GSE33315, GSE7440, GSE11877, and GSE13204, and TCGA database (https://cancergenome.nih.gov/).

## Author Contributions

SZ and ZL designed the study. XiY, YD, FZ, PW, JZ, DY, FL, XZ, and DZ wrote the manuscript. LB, XX, HW, XuY, WaZ, HG, and WeZ performed the data analyses. LY, QH, HX, YS, YZ, and YW contributed to the conception of the study and drafted the manuscript. All authors contributed significantly in writing the manuscript and read and approved the final manuscript.

## Conflict of Interest Statement

The authors declare that the research was conducted in the absence of any commercial or financial relationships that could be construed as a potential conflict of interest.

## Footnotes

1 https://string-db.org/

2 http://david.abcc.ncifcrf.gov/

3 http://geneontology.org/

4 http://jaspar.genereg.net/

5 http://cancer.sanger.ac.uk/cosmic

6 http://bioinfo.life.hust.edu.cn/AnimalTFDB2/

7 https://xenabrowser.net
