# Supplementary figures and images for: Regulatory Network and Prognostic Effect Investigation of PIP4K2A in Leukemia and Solid Cancers
Source: Front Genet. 2019 Jan 15;9:721. doi: 10.3389/fgene.2018.00721 (PMC6341070; doi:10.3389/fgene.2018.00721)

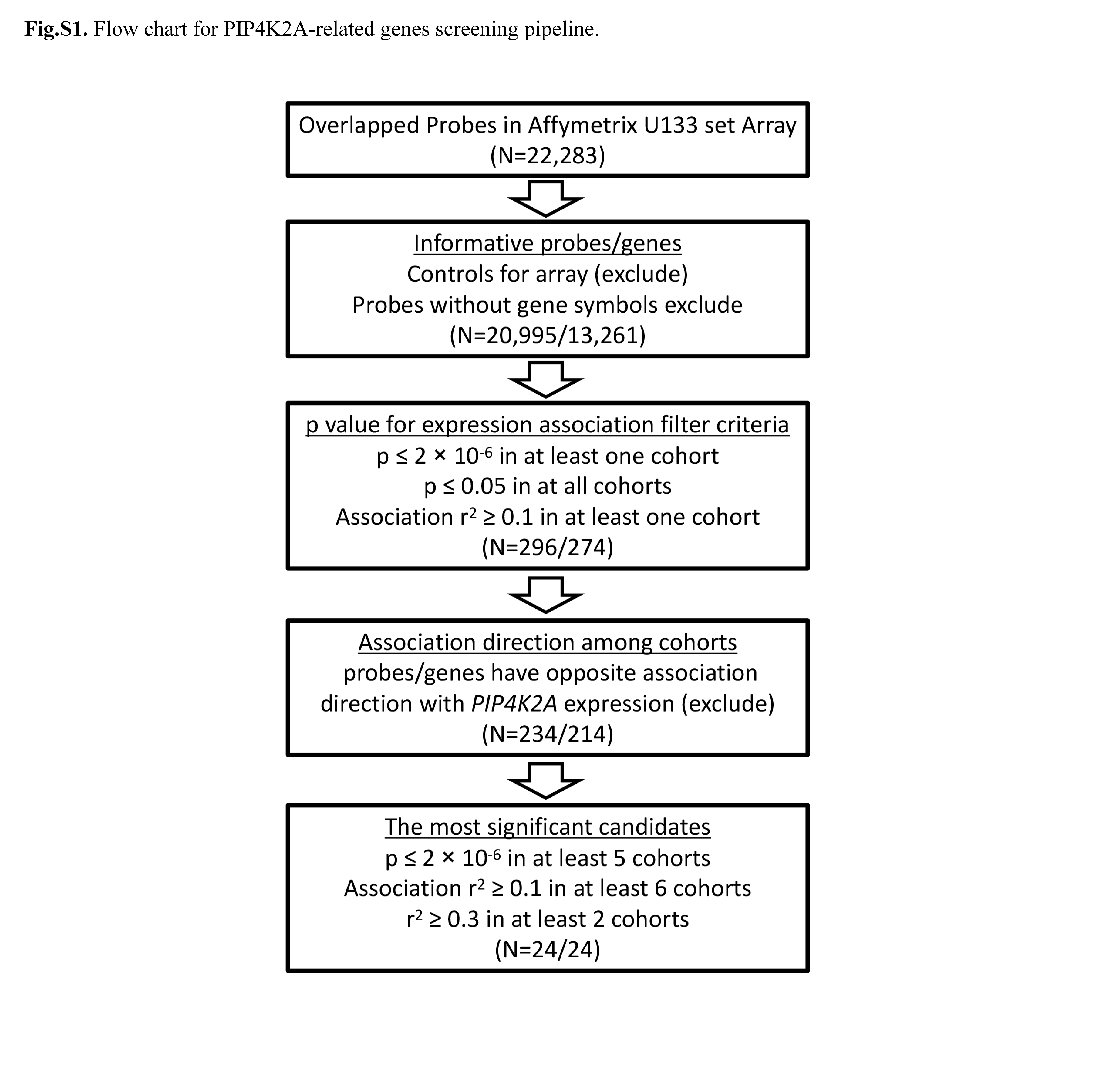

Supplement: Supplementary file 1 [file Image_1.TIF]

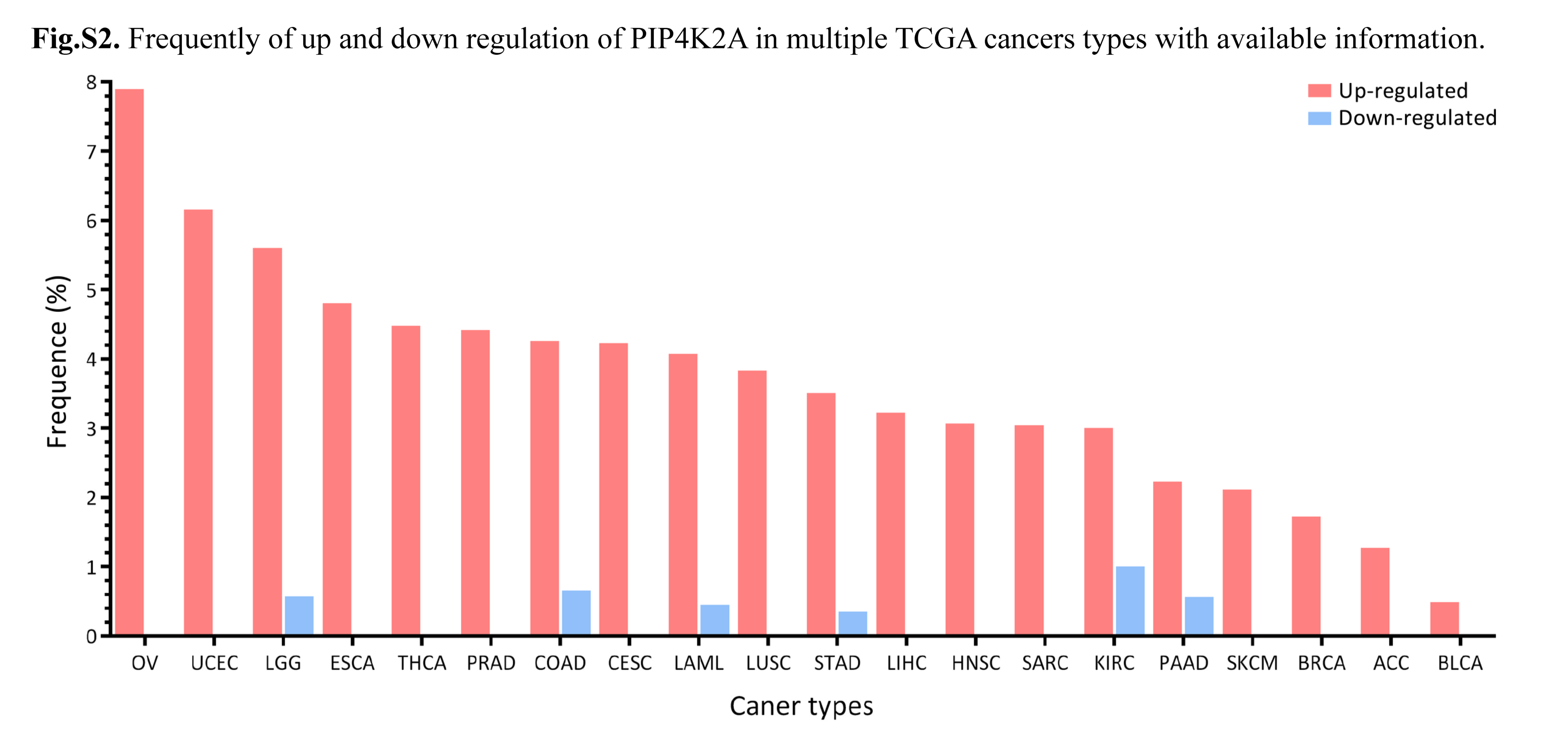

Supplement: Supplementary file 2 [file Image_2.TIF]

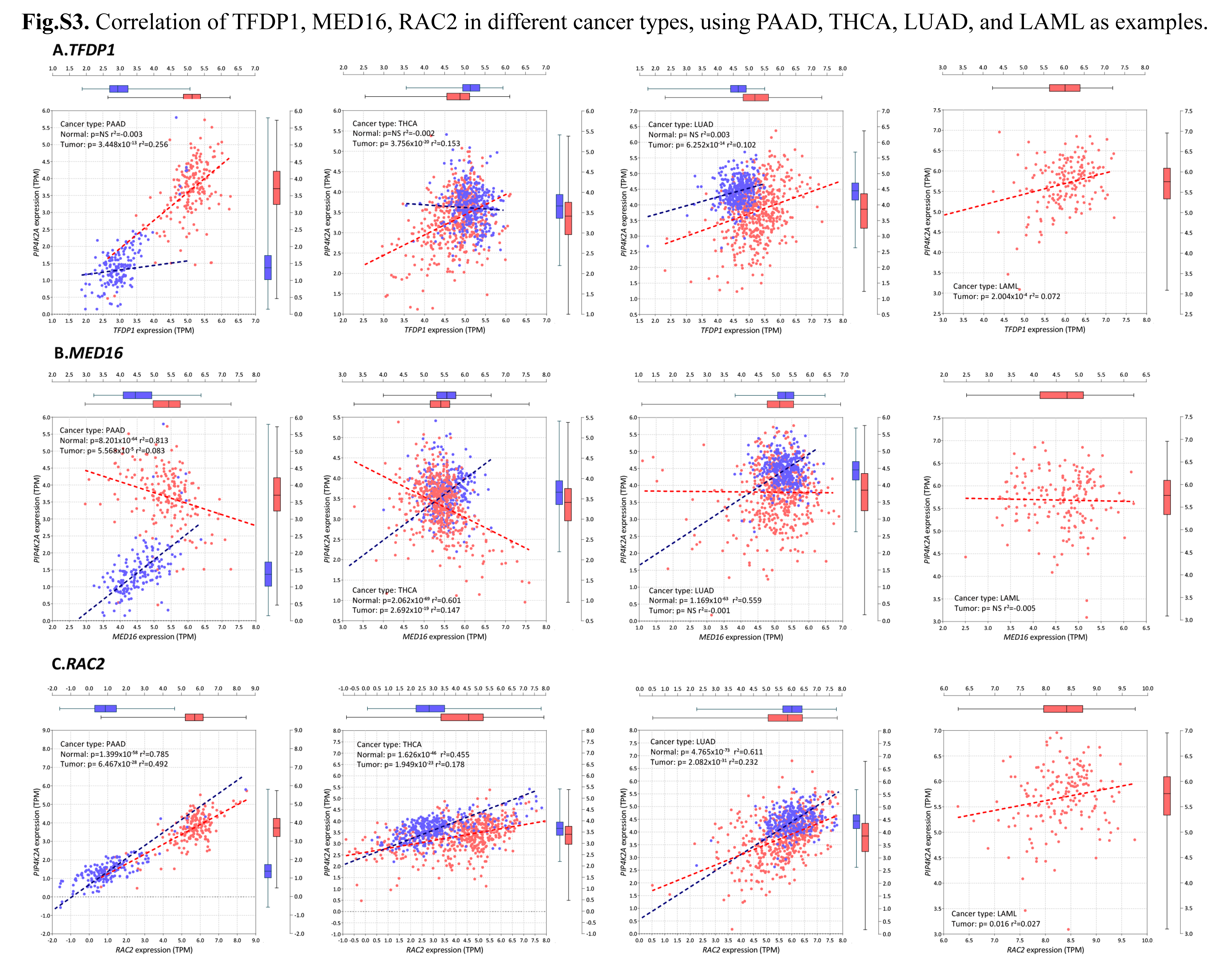

Supplement: Supplementary file 3 [file Image_3.TIF]

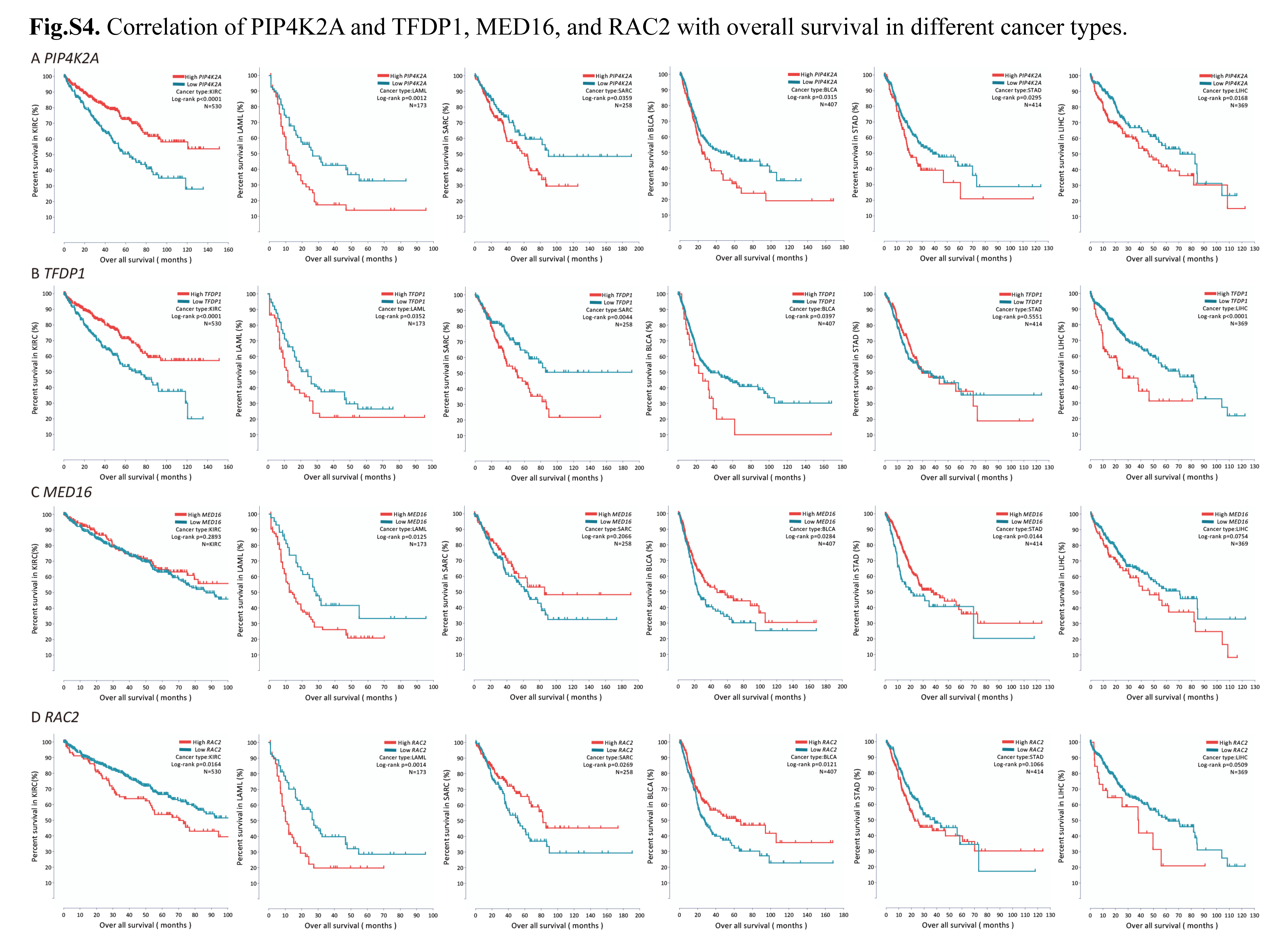

Supplement: Supplementary file 4 [file Image_4.TIF]

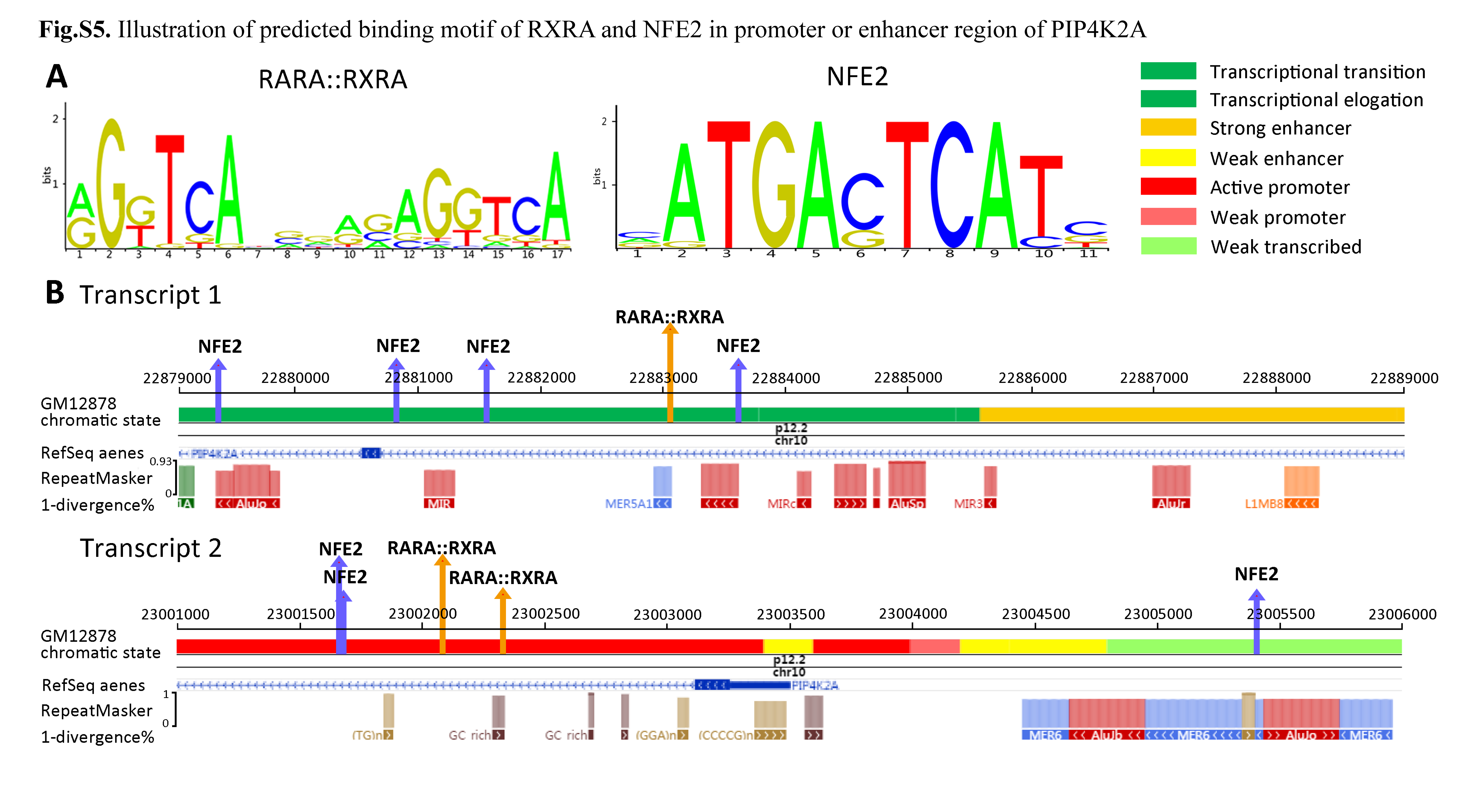

Supplement: Supplementary file 5 [file Image_5.TIF]

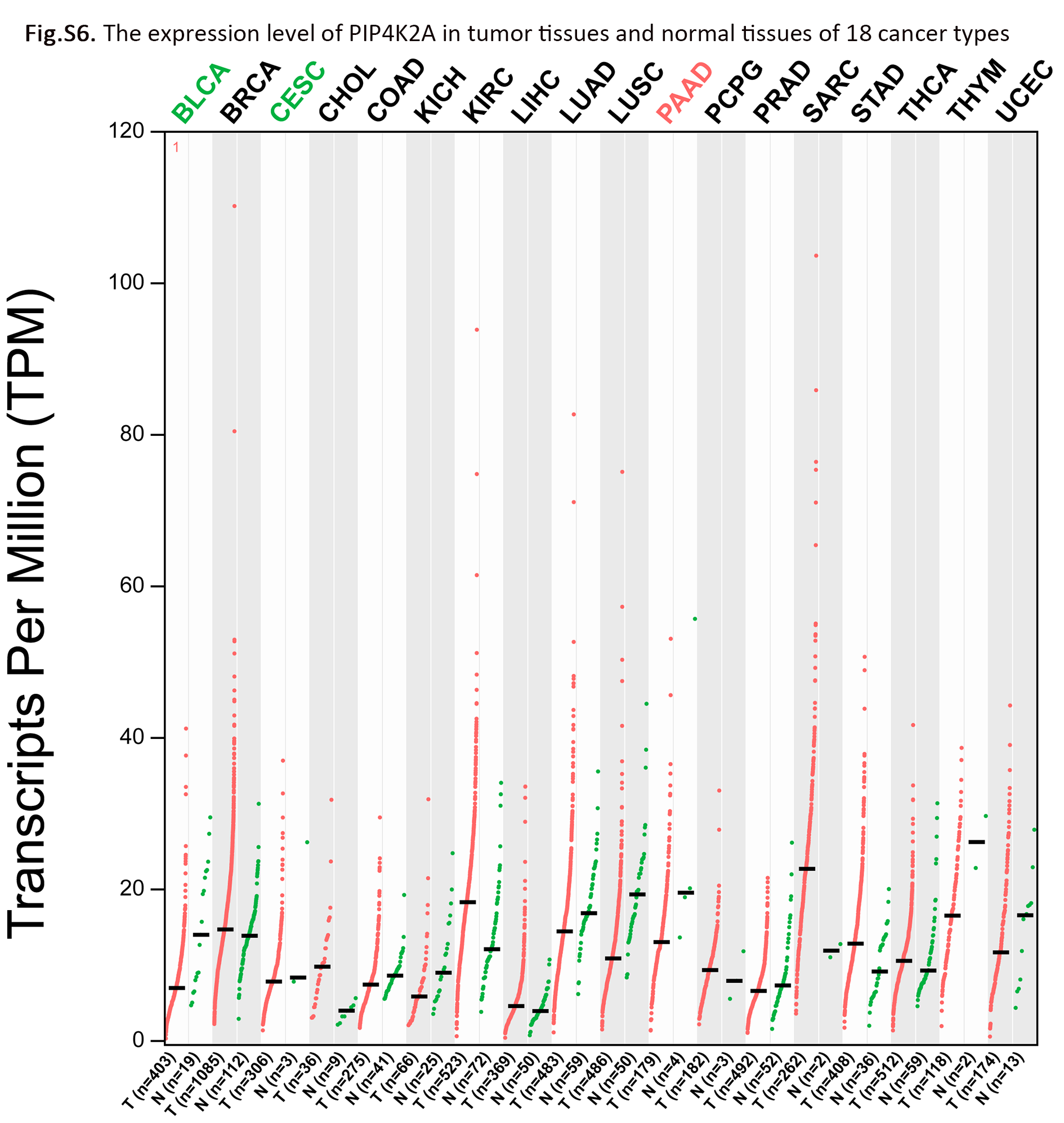

Supplement: Supplementary file 6 [file Image_6.tif]
